# Health benefits of physical activity for people with mental disorders: From the perspective of multidimensional subjective wellbeing

**DOI:** 10.3389/fpsyt.2022.1050208

**Published:** 2022-11-17

**Authors:** Chao Li, Guangjie Ning, Yuxin Xia, Qianqian Liu

**Affiliations:** ^1^Business School, Shandong University, Weihai, China; ^2^HSBC Business School, Peking University, Shenzhen, China

**Keywords:** physical activity, people with depression, health benefits, multidimensional subjective wellbeing, psychiatric treatment

## Abstract

This paper uses a large scale and nationally representative dataset, Chinese General Social Survey, to empirically examine the role of physical activity in reducing the negative effects of depression among people with mental disorders. Empirical results demonstrate that physical exercise could help to alleviate depression's adverse consequences on work and life for depressed individuals. The impact mechanism is that physical activity may decrease the severity of depression, enhance life satisfaction, improve mood, and make people have a better sense of purpose and meaning in life. Therefore, from the perspective of multidimensional subjective wellbeing, evaluative wellbeing, experienced wellbeing and eudaimonic wellbeing all play mediating roles in the reduction of depression's adverse effects. Heterogeneity analysis shows that there are no significant gender differences in the health benefits of physical exercise, but its impact tends to be more prominent for depressed individuals who are younger and higher educated, with better health status, and live in urban areas. It is also found that socioeconomic status may play an important moderating role. The health benefits of physical activity seem to be greater for depressed people who have lower income, work in the secondary labor market, and have lower levels of social capital and assets. In addition, the instrumental variable approach is used to identify the causal impact of physical activity, which further proves a significant effect of it based on tackling the endogeneity problem. Meanwhile, this paper uses different explanatory and explained variables, different statistical models, as well as machine learning and placebo techniques to conduct robustness tests, all of which lend credence to above findings.

## Introduction

Mental health awareness has taken hold over the recent years and psychiatric health is widely regarded as important as physical health (1). Depression is a typical manifestation of mental disorders, decreasing people's quality of life and working ability. Studies have shown that depression is strongly associated with lower life satisfaction, poorer overall health and even higher risk of death (2–4). At the same time, those with mental disorders are faced with more difficulties to obtain job opportunities in the labor market, forcing them to accept lower wages and thus bear greater financial burdens, which in turn exacerbates depressive disorders (5). In addition, depression brings about stigma to patients due to misconceptions in the public. Such an effect not only causes them to feel humiliated, but also drives them out of social contacts (6). Therefore, effective treatment of depression is very essential for improving people's mental health and achieving the goal in the United Nations' *2030 Agenda for Sustainable Development*, that is “ensuring healthy lives and promoting wellbeing for all” (7).

Existing studies have examined the effectiveness of multiple treatments for mental disorders, such as antidepressants (8), psychotherapy (9, 10), and lifestyle improvement (3, 4). Although medication is effective and necessary in many cases, studies have shown that antidepressants bring about notable side effects (11, 12). Therefore, it is necessary to explore alternative solutions for the treatment of mental disorders. In recent years, there has been growing interest in whether physical activity contributes to the welfare of people with depression. Physical activity is believed to have fewer side effects and is widely accepted, with significant correlations with both physical and mental health (13–19). In terms of mental health, maintaining a regular exercise routine can improve people's healthy living habits and help them reduce emotional exhaustion (15). Studies have shown a correlation between physical activity and mental health, and that physical exercise may reduce the risk of depression to some extent (20–22). In terms of the duration of physical activity, research shows that 3–5 times per week of moderate-intensity training is sufficient to reduce depression levels (4, 23). In recent years, COVID-19 has increased depressive disorders as people have less opportunity to participate in exercise due to isolation and limited social activities (16, 24). Existing studies have examined the effect of physical exercise in impacting people's health status during the epidemic both psychologically and physiologically (17, 23). In addition, it is found that team sports increase the frequency of interpersonal interactions, which can have a positive effect on mental health (18, 19). This echoes findings that physical activity helps people effectively deal with negative emotions caused by limited interactions (25). Furthermore, physical activity can also improve people's physical and cognitive abilities, thereby improving their capability to cope with negative emotions (26). Subjective wellbeing is also an important factor affecting mental health (27) and research has demonstrated that exercise contributes to people's wellbeing (28). The main reason for this is the positive effect of exercise on body image (29), and this conclusion holds for various types of physical activities (30).

In addition, existing studies have shown that there may exist heterogeneities in the effects of physical activity across different groups. As regards sociodemographic characteristics, it is found that exercise's effect is heterogeneous in terms of gender and age (31, 32). For example, regular exercise is an effective way to maintain a good psychological state and its benefits are more prominent for older people (33). Among the elderly, moderate exercise is very helpful in promoting mental health (34, 35). In addition, women exhibit higher levels of depression and therefore are more likely to gain health benefits from regular exercises (17, 32). Compared to men's preference for vigorous exercise, women are more likely to engage in low-intensity activities, such as walking, jogging and yoga (36). For example, square dancing, which is widely practiced among older people in China, can effectively reduce mental disorders (37). In terms of personality traits, people with high self-efficacy are generally more active in sports and mentally healthier (38). With regards to occupational characteristics, it is found that jobs that require long periods of sedentary work are more likely to cause anxiety and depression in workers, and physical activity has a more significant effect on them (39).

Compared with the existing research, the significance and value of this paper are mainly reflected in three aspects. First, this paper confirms the benefits of physical activity for people with mental disorders. Existing studies indicate that there is a correlation between physical activity and depression (20–22), but systematic empirical tests are still needed concerning whether exercise weakens the adverse effects of depression on the life and work among people with mental disorders. Specifically, the causal effect of physical activity awaits scientific examination. Second, this paper detects the mechanisms by which physical activity exerts its positive effect on people with mental disorders from multidimensional wellbeing. In terms of how exercises affect mental disorders, existing research suggests the relationship between exercises and subjective wellbeing (28–30), as well as associations between mental health and wellness (27). However, it remains to be investigated whether physical activity reduces the negative effects of depression by improving people's subjective wellbeing. Third, this paper systematically examines the heterogeneity of benefits brought by physical activity. Literature demonstrates variations in exercise habits among people with different characteristics (31–39), but it is unclear whether there also exist heterogeneities in the health benefits of physical activity. Based on this, this paper systematically examines whether physical exercise brings health benefits to people with depressive disorders using a large-scale representative micro dataset in China, and conducts mechanism analysis from the perspective of multidimensional subjective wellbeing. In addition, this research deals with endogeneity to test causality using the instrumental variable approach, and conducts heterogeneity analysis as well as robustness checks in multiple aspects.

## Materials, measures, and methods

### Data source

Data used in this research is the Chinese General Social Survey (CGSS) from 2017 to 2018, which is one of the most important national microdata in China. CGSS is in the world General Social Survey family, mainly carried out by Renmin University of China. CGSS aims to systematically and comprehensively reflect living conditions of Chinese people. Detailed information of CGSS is provided in Supplementary materials and can also be accessed through http://cgss.ruc.edu.cn/English/Home.htm. The reasons for using CGSS in this paper are mainly due to three aspects. First, CGSS asks respondents whether they are depressed and the extent to which depression adversely affects their lives and work. It also comprehensively contains factors relevant to mental health discussed in the existing literature. This not only enables us to construct control variables, but also facilitates an in-depth heterogeneity analysis. Second, CGSS examines people's exercise habits, which facilitates the construction of explanatory variables for this study. Third, CGSS includes different dimensions of people's subjective wellbeing in the extension module including evaluative wellbeing, experienced wellbeing and eudaimonic wellbeing. This allows us to systematically examine physical activity's impact mechanisms from the perspective of multidimensional subjective wellbeing.

### Measures

Participants of this research are those who report that they suffer from depressive disorders in CGSS. The main explained variable is the extent to which people's life and work are negatively affected by depression, denoted as *Problem*_*depression*. This variable is based on the Likert scale to classify the severity of depressive disorders, where points from 1 to 5 represent depression never, seldom, sometimes, often and always exerting negative effects on their life and work. The explanatory variable in this paper is the frequency of participating in physical activity, denoted as *Physical*_*activity*. This variable comes from the question: “In the past 12 months, how many times did you usually engage in physical activity that lasts at least 30 min and makes you sweat per week?”.

Based on literature concerning depressive disorders and its consequences on people's lives and work (1–3, 6, 10, 15), in order to avoid the omitted variable bias, this paper comprehensively controls factors related to effects of depression, including variables of demographic characteristics, human capital characteristics, social characteristics, working characteristics, family characteristics, and regional and time characteristics. (1) Demographic characteristics include age, the squared term of age and gender. (2) Human capital characteristics include educational level and whether the respondent is a migrant. (3) Social characteristics include whether her/his Hukou^1^ is in urban, whether being ethnic minorities, whether having religious beliefs and whether being the Communist Party of China (CPC) member. (4) Working characteristics include personal income, whether working in the system^2^ and whether having pension and medical insurance. (5) Family characteristics include marital status, family size, number of children and number of housing assets. (6) Regional and time features include provincial and year dummies. The descriptive statistics results of above variables are shown in Supplementary Table 1.

### Methods

Because the explained variable, which is the extent to which life and work are adversely affected by depression, is an ordered variable, we use the Ordered Probit model to conduct regression. Specifically, based on *Problem*_*depression*_*i*_, the sample is divided into 5 different groups. Groups *g* = 1 to 5 represent those whose life and work are never, seldom, sometimes, often and always negatively affected by depression, respectively. The probability of a given observation *i* for the Ordered Probit model *p*_*gi*_ is


$$\begin{array}{l} p_{gi}\;=\text{ Pr}\left(Problem\_depression_{i}\;=\;g\right)\;=\text{ Pr}(\chi_{g-1}<\alpha \\ \;+\beta Physical\_activity_{i}+x_{i}^{'}\gamma +\varepsilon_{i}\leq \chi_{g})\\ \;=\;\Phi (\chi_{g}-\alpha -\beta Physical\_activity_{i}-x_{i}^{'}\gamma )-\Phi (\chi_{g-1}\\ \;-\;\alpha \;-\;\beta Physical\_activity_{i}\;-\;x_{i}^{'}\gamma ) \end{array}$$

where *Problem*_*depression*_*i*_ and *Physical*_*activity*_*i*_ are the dependent and explanatory variables, $x_{i}^{'}$ is a vector of controls introduced above, χ_0_ is −∞, χ_5_ is +∞ and Φ(·) is the standard normal cumulative distribution function. Accordingly, the log likelihood of the maximum likelihood estimation (MLE) is


$$\begin{array}{l} lnL\;=\;\overset{N}{\underset{i=1}{\sum }}\overset{5}{\underset{g=1}{\sum }}I_{g}\left({Problem\text{\_}depression}_{i}\right)\;ln\;p_{gi} \end{array}$$

where $I_{g}(Problem\_depression_{i})=\{\begin{array}{c} 1,\;if\;Problem\_depression_{i}=g\\ 0,\;if\;Problem\_depression_{i}\neq g \end{array}$ and *N* is the sample size. Based on this, β and **γ** are estimated by max*lnL*. The data analysis software used in this paper is Stata 17.0.

## Results

### Benchmark results

Table 1 shows the regression results by the above Ordered Probit model. Column (1) is the estimation without including any control variables, showing that *Physical*_*activity* is significantly and negatively correlated with *Problem*_*depression*. In Columns (2)–(6), control variables of demographic characteristics, human capital characteristics, social characteristics, working characteristics, family characteristics, and regional and time characteristics are sequentially added into the regressions. Results demonstrate that the estimated coefficients of physical activity are significantly negative at the 1% level in all regressions. It means that for people with mental disorders, the higher the frequency of physical exercise, the less their work and daily life are adversely affected by depression problems. In addition, the estimated coefficient of physical activity decreases slightly but remains very stable as different aspects of characteristics are controlled. This shows that the significant relationship between *Physical*_*activity* and *Problem*_*depression* can hardly be affected by other factors and is very robust. The estimates of control variables are basically in line with theoretical expectations and are consistent with the existing research. In terms of gender, women are more likely to suffer from depression in their work and life, consistent with findings in Lyttelton et al. (40). The estimated coefficient of the squared term of age is significantly negative, while that of age is positive. This implies an upward trend in depression symptoms as people get older and a negative relationship after a turning point (41, 42). In terms of working characteristics, lower income level and inadequate social security will increase the adverse impacts of mental disorders. In addition, married individuals have lower levels of depression, especially among those with higher marital quality (43, 44). Raising children requires people to bear more time and financial costs, so the greater the number of children, the more prominent the problems in terms of mental disorders (45).

**Table 1:** Benchmark regression results.

**Model**	**(1) Oprobit**	**(2) Oprobit**	**(3) Oprobit**	**(4) Oprobit**	**(5) Oprobit**	**(6) Oprobit**	**(7) Oprobit**
**Variable**	**Problem_ depression**	**Problem_ depression**	**Problem_ depression**	**Problem_ depression**	**Problem_ depression**	**Problem_** **depression**	**Problem_** **depression**
Physical_ activity	−0.042^***^ (0.003)	−0.042^***^ (0.003)	−0.040^***^ (0.003)	−0.034^***^ (0.003)	−0.033^***^ (0.003)	−0.033^***^ (0.003)	−0.031^***^ (0.003)
Age		0.039^***^ (0.003)	0.032^***^ (0.003)	0.032^***^ (0.003)	0.038^***^ (0.003)	0.045^***^ (0.003)	0.042^***^ (0.003)
Age_squared		−0.000^***^ (0.000)	−0.000^***^ (0.000)	−0.000^***^ (0.000)	−0.000^***^ (0.000)	−0.000^***^ (0.000)	−0.000^***^ (0.000)
Whether female		0.114^***^ (0.016)	0.112^***^ (0.016)	0.100^***^ (0.016)	0.061^***^ (0.017)	0.056^***^ (0.017)	0.075^***^ (0.018)
Education level			−0.177^***^ (0.029)	−0.027 (0.031)	−0.002 (0.033)	0.005 (0.034)	0.047 (0.034)
Whether migrants			−0.245^***^ (0.026)	−0.239^***^ (0.026)	−0.219^***^ (0.028)	−0.232^***^ (0.028)	−0.127^***^ (0.030)
Whether Hukou in urban				−0.236^***^ (0.018)	−0.184^***^ (0.019)	−0.183^***^ (0.020)	−0.111^***^ (0.021)
Whether ethnic minorities				0.145^***^ (0.032)	0.143^***^ (0.033)	0.142^***^ (0.033)	0.061 (0.038)
Whether religious believer				−0.054^*^ (0.028)	−0.050^*^ (0.029)	−0.047 (0.029)	0.012 (0.030)
Whether CPC member				−0.192^***^ (0.030)	−0.155^***^ (0.031)	−0.135^***^ (0.031)	−0.130^***^ (0.031)
ln_Income					−0.028^***^ (0.002)	−0.028^***^ (0.002)	−0.024^***^ (0.002)
Whether working in the system					0.003 (0.038)	0.003 (0.038)	−0.010 (0.039)
Whether having pension					−0.048^**^ (0.021)	−0.040^*^ (0.021)	−0.010 (0.021)
Whether having medical insurance					0.061^*^ (0.035)	0.087^**^ (0.036)	0.071^**^ (0.036)
Whether married						−0.115^***^ (0.022)	−0.112^***^ (0.023)
Family size						−0.028^***^ (0.006)	−0.027^***^ (0.006)
Number of children						0.027^***^ (0.008)	0.017^**^ (0.008)
Number of houses						−0.089^***^ (0.015)	−0.075^***^ (0.015)
Year dummies	No	No	No	No	No	No	Yes
Province dummies	No	No	No	No	No	No	Yes
Observations	17,840	17,840	17,786	17,740	16,721	16,559	16,559
Pseudo R^2^	0.005	0.058	0.061	0.066	0.068	0.070	0.079

The values in parentheses are standard errors robust to heteroskedasticity. Yes means the corresponding variables are controlled in the regression, while No means not controlled.

***, **, and * indicate significance at the levels of 1, 5, and 10%, respectively.

### Mechanism analysis

Studies have shown that physical activity helps to increase people's enthusiasm for life and improve their subjective wellbeing (28–30). The question naturally arises that whether physical exercise reduces the extent to which people suffer from depression by reducing the severity of depression and improving the subjective wellbeing. To test this hypothesis, firstly, we use respondents' answers to the question “To what extent do you feel depressed?” to characterize the severity of depression to conduct the mechanism analysis. Responses to this question include “not depressed,” “mildly depressed,” “moderately depressed,” “very depressed” and “severely depressed.” Second, based on the measures of multidimensional subjective wellbeing in the existing literature (46–48), we test the mediating roles of evaluation wellbeing (life satisfaction), experienced wellbeing (the emotions that people experience in their lives) and eudaimonic wellbeing (sense of purpose and meaning in life). The variable of Evaluative wellbeing comes from the respondents' degree of agreement on “In general, how are you satisfied with your life?”. Experienced wellbeing_1, Experienced wellbeing_2 and Experienced wellbeing_3 come from the respondents' degree of agreement on “I feel that the society is providing more and more opportunities for people to develop themselves,” “I am content with my life compared to others around me” and “I am satisfied with my family's income level,” respectively. Furthermore, Eudaimonic wellbeing_1 , Eudaimonic wellbeing_2 and Eudaimonic wellbeing_3 come from the respondents' degree of agreement on “I have goals in life and they motivate me to live better,” “I am currently doing my best to pursue my goals in life” and “When things are uncertain, I usually expect them to go in the good direction” respectively. These questions are all based on the 5-Point Likert Scale, with higher values representing higher levels of wellbeing. The Cronbach's Alpha for the multidimensional sub-scales of subjective wellbeing is 0.891, indicating good internal consistency.

Results of the mechanism analysis using above variables are shown in Table 2. Column (1) demonstrates that physical activity helps reduce the severity of depression among people with mental disorders. Besides, other odd-numbered columns in Table 2 display that physical exercise has a significant positive effect on depressed individuals' subjective wellbeing in all of the three dimensions. This means that the more frequently they participate in exercises, the higher their wellness will be, including the evaluative wellbeing, experienced wellbeing and eudaimonic wellbeing. Specifically, it is demonstrated that exercises help to increase the life satisfaction of people with depressive disorders, improving emotions in their lives and helping them have a better sense of purpose and meaning in life. Results of even-numbered columns in Table 2 show that when the mediators are included in the regressions, their estimated coefficients are all significantly positive at the 1% level. This suggests that multidimensional subjective wellbeing plays a mediating role in the health benefits of exercise for people with depression. Therefore, participation in physical activity alleviates the adverse effects of mental disorders on life and work by both decreasing depression levels and increasing people's subjective wellbeing. It is worth pointing out that the sample size is smaller in the regressions testing multidimensional subjective wellbeing since relevant questions come from the extension module, where only some of the randomly selected respondents are asked to answer questions therein. Despite the sample loss, it is obvious that all of these mechanism analysis results are significant and the conclusions are robust.

**Table 2:** Mechanism analysis.

**Model**	**(1)Oprobit**	**(2)Oprobit**	**(3)Oprobit**	**(4)Oprobit**	**(5)Oprobit**	**(6)Oprobit**	**(7)Oprobit**	**(8)Oprobit**
**Variable**	**Depression**	**Problem_** **depression**	**Evaluative wellbeing**	**Problem_** **depression**	**Experienced wellbeing_1**	**Problem_** **depression**	**Experienced wellbeing_2**	**Problem_** **depression**
Physical_ activity	−0.018^***^ (0.003)	−0.027^***^ (0.003)	0.025^***^ (0.008)	−0.037^***^ (0.008)	0.015^**^ (0.007)	−0.038^***^ (0.1008)	0.036^***^ (0.007)	−0.035^***^ (0.008)
Depression		0.452^***^ (0.013)						
Evaluative wellbeing				−0.194^***^ (0.026)				
Experienced wellbeing_1						−0.047^**^ (0.024)		
Experienced wellbeing_2								−0.057^**^ (0.023)
Controls	Yes	Yes	Yes	Yes	Yes	Yes	Yes	Yes
Observations	16,579	16,559	2,734	2731	2,678	2,675	2,681	2,678
Pseudo R ^2^	0.028	0.111	0.039	0.107	0.027	0.101	0.029	0.101
**Model**	**(9) Oprobit**	**(10) Oprobit**	**(11) Oprobit**	**(12) Oprobit**	**(13) Oprobit**	**(14) Oprobit**	**(15) Oprobit**	**(16)Oprobit**
**Variable**	**Experienced wellbeing_3**	**Problem_** **depression**	**Eudaimonic wellbeing_1**	**Problem_** **depression**	**Eudaimonic wellbeing_2**	**Problem_** **depression**	**Eudaimonic wellbeing_3**	**Problem_** **depression**
Physical_ activity	0.045^***^ (0.007)	−0.034^***^ (0.008)	0.024^***^ (0.007)	−0.036^***^ (0.008)	0.036^***^ (0.007)	−0.035^***^ (0.008)	0.019^***^ (0.007)	−0.039^***^ (0.008)
Experienced wellbeing_3		−0.107^***^ (0.018)						
Eudaimonic wellbeing_1				−0.108^***^ (0.024)				
Eudaimonic wellbeing_2						−0.050^***^ (0.016)		
Eudaimonic wellbeing_3								−0.068^**^ (0.033)
Controls	Yes	Yes	Yes	Yes	Yes	Yes	Yes	Yes
Observations	2,707	2,704	2,643	2,640	2,626	2,623	2,710	2,707
Pseudo R^2^	0.041	0.104	0.030	0.103	0.061	0.102	0.039	0.101

*** and ** indicate significance at the levels of 1, 5, and 10%, respectively.

### Heterogeneities analysis

In order to further examine the heterogeneous effects of *Physical*_*activity*, we conduct moderating effects analysis in multiple aspects. Regression results are shown in Table 3. Here we primarily focus on the estimated coefficients of the interactions between the moderating variables and physical activity. If the interaction term's estimate is significantly negative, it means that the moderator would increase the health benefits of physical exercise in reducing depression's adverse effect. Otherwise, the opposite result would mean that the factor reduces physical activity's impact.

**Table 3:** Heterogeneities analysis.

**Model**	**(1) Oprobit**	**(2) Oprobit**	**(3) Oprobit**	**(4) Oprobit**	**(5) Oprobit**	**(6) Oprobit**
**Moderator variable**	**Whether female**	**Whether younger than 50**	**Education level**	**Health condition**	**Whether Hukou in urban**	**Whether religious believer**
Interaction between physical_activity and moderator	0.002 (0.006)	0.033^***^ (0.006)	0.002^**^ (0.001)	0.017^***^ (0.003)	−0.013^**^ (0.006)	−0.013 (0.010)
Physical activity	−0.031^***^ (0.004)	−0.045^***^ (0.004)	−0.039^***^ (0.005)	−0.072^***^ (0.010)	−0.024^***^ (0.004)	−0.029^***^ (0.003)
Moderator variable	0.072^***^ (0.021)	−0.617^***^ (0.024)	−0.030^***^ (0.005)	−0.725^***^ (0.012)	−0.116^***^ (0.024)	0.039 (0.036)
Other controls	Yes	Yes	Yes	Yes	Yes	Yes
Observations	16,559	16,559	16,559	16,554	16,559	16,559
Pseudo R^2^	0.080	0.070	0.080	0.190	0.080	0.080
**Model**	**(7) Oprobit**	**(8) Oprobit**	**(9) Oprobit**	**(10) Oprobit**	**(11) Oprobit**	**(12) Oprobit**
**Moderator variable**	**Whether ethnic minorities**	**ln_Family_** **income**	**Whether working in the system**	**Social capital**	**Whether owning cars**	**Whether having commercial medical insurance**
Interaction between physical_activity and moderator	−0.017 (0.013)	0.003^**^ (0.001)	0.023^**^ (0.010)	0.019^***^ (0.004)	0.021^***^ (0.006)	0.027^***^ (0.010)
Physical activity	−0.029^***^ (0.003)	−0.060^***^ (0.015)	−0.033^***^ (0.003)	−0.056^***^ (0.006)	−0.036^***^ (0.004)	−0.033^***^ (0.003)
Moderator variable	0.088^**^ (0.043)	−0.057^***^ (0.005)	−0.147^***^ (0.041)	−0.073^***^ (0.017)	−0.194^***^ (0.026)	−0.169^***^ (0.040)
Other controls	Yes	Yes	Yes	Yes	Yes	Yes
Observations	16,559	15,843	16,559	16,537	16,547	16,452
Pseudo R^2^	0.080	0.080	0.080	0.080	0.080	0.080

Other controls include all the controls other than the moderator variable. In column (8), personal income is not controlled to avoid multicollinearity as family income is controlled as the moderator variable.

*** and ** indicate significance at the levels of 1, 5, and 10%, respectively.

First, we examine the heterogeneities in terms of two demographic characteristics, which are gender and age. With regard to gender, results in column (1) of Table 3 show that the interaction term between gender and physical exercise is not significant, meaning that there is no obvious difference in *Physical*_*activity*'s role to lower *Problem*_*depression* for men and women. In respect of age, column (2) of Table 3 demonstrates that if the depressed individuals are under the age of 50, the effect of physical activity on reducing depression's consequences is more prominent. This may be due to the fact that younger individuals are more physically active and more likely to engage in physical activities with higher intensity and longer duration. Therefore, the health benefits of physical exercise for younger depressed people are more prominent. Furthermore, columns (3) and (4) show that, in terms of human capital characteristics, the interaction terms of both education level and health status with *Physical*_*activity* are significantly positive^3^. This demonstrates that rewards from exercise are greater for those with higher levels of human capital. In addition, regarding social characteristics, column (5) shows that the health benefits of physical activity are greater for depressed residents in urban areas, which is consistent with the theoretical expectations because there are more sports venues and better sports infrastructure in urban areas. Thus, compared with their rural counterparts, urban residents have more access to professional physical exercises, which is more helpful to reduce the adverse effects of depression on their life and work. However, results in columns (6) and (7) indicate that whether the individuals are religious believers and whether they belong to ethnic minorities do not alter physical activity's benefits.

It is also found that socioeconomic status plays an important moderating role in the relationship between *Physical*_*activity* and *Problem*_*depression*. Specifically, we examine the heterogeneities in aspects of income, job characteristics, social capital, assets, and social security among depressed individuals. First, results in column (8) of Table 3 show that physical activity alleviates the negative effects caused by depression in the lower-income subgroup more prominently. Besides, working in-system in China refers to having jobs in Communist Party of China organizations, governments and state-owned corporations. Working in these sectors is perceived as having higher social status and social capital. Therefore, in-system work is traditionally regarded as the primary labor market in China, while out-of-system means the secondary labor market. We conduct heterogeneity analysis from the perspective of social status according to this employment characteristic. Meanwhile, we use respondents' answers to “how often you use your work to help your family and friends” (ranging from 1 to 5) as an indicator of social capital. Results of columns (9) and (10) demonstrate that for depressed individuals who work outside the system and whose social capital is lower, the role of physical activity in reducing depression's detrimental effects is more pronounced. In addition, columns (11) and (12) indicate that the effect of exercise is greater for depressed people not owning cars and commercial medical insurance. Overall, heterogeneity analysis in terms of socioeconomic status reveals that for depressed individuals with lower socioeconomic status, *Physical*_*activity* has a greater effect in reducing *Problom*_*depression*. The reason behind it may be attributed to the fact that the negative impact of depression on work and life is significantly higher for groups with lower socioeconomic status. For example, the averages of *Problom*_*depression* among depressed respondents with lower incomes and working outside the system are, respectively, 2.49 and 2.13, which are significantly higher than their counterparts, which are 1.86 and 1.62, respectively. Therefore, the health benefits gained from physical activity for them tend to be greater.

### Dealing with endogeneity

To examine the causal effect of physical activity on reducing the adverse effect of depression for people with mental disorders, this paper uses the instrumental variable method to tackle the endogeneity problem. Specifically, we construct the following two-stage least squares (2SLS) statistical model, applying the degree of automation's replacement of tasks in work as the instrumental variable.


$$\begin{array}{l} \;Physical\_activity_{i}=\;\;\gamma_{0}\;+\;\gamma_{1}Automation_{i}\;+\;x_{i}^{{}^{\prime }}\psi^{1}\;+\;\mu_{i}^{1}\\ Problem\_depression_{i}=\;\;\delta_{0}\;+\;\delta_{1}\hat{Physical\_activity_{i}}\;+\;x_{i}^{{}^{\prime }}\psi^{2}\\ \;+\;\mu_{i}^{2} \end{array}$$

In the model, $x_{\text{i}}^{'}$ is a vector of a set of control variables described above. *Automation*_*i*_ is the automation indicator, which is the instrumental variable constructed by Mihaylov and Tijden (49). This measure is the extent to which the tasks in individual *i*'s occupation are replaced by automation. The higher this index, the fewer time people need to spend in working (50) and thus the more time they can participate in sports. Therefore, this instrumental variable satisfies the correlation prerequisite. Statistical tests on this are shown in Supplementary Table 2. Furthermore, automation depends on exogenous technology change, independent of the micro individual's personal characteristics. Consequently, this instrumental variable meets the requirement of exogeneity. Regression results are shown in Table 4, and due to space limitations the full table of estimations is shown in Supplementary Table 3. It is noted that 2SLS estimates with the instrumental variable are basically consistent with that of benchmark regressions. With gradually adding different characteristics, estimated coefficients of physical activity are all significantly negative at the 1% level. This means that the health benefits of physical activity for depressed people are not subject to endogeneity problems. Results using other IV approaches demonstrate consistent findings with that applying 2SLS and are provided in Supplementary Table 4.

**Table 4:** Endogeneity treatment: instrumental variable regressions.

**Model**	**(1) 2SLS**	**(2) 2SLS**	**(3) 2SLS**	**(4) 2SLS**	**(5) 2SLS**	**(6) 2SLS**	**(7) 2SLS**
**Variable**	**Problem_** **depression**	**Problem_** **depression**	**Problem_** **depression**	**Problem_** **depression**	**Problem_** **depression**	**Problem_** **depression**	**Problem_** **depression**
Physical_activity	−0.846^***^ (0.115)	−0.450^***^ (0.077)	−0.437^***^ (0.082)	−0.473^***^ (0.128)	−0.384^***^ (0.110)	−0.402^***^ (0.122)	−0.378^***^ (0.138)
Demographic characteristics	N	Y	Y	Y	Y	Y	Y
Human capital characteristics	N	N	Y	Y	Y	Y	Y
Social characteristics	N	N	N	Y	Y	Y	Y
Working characteristics	N	N	N	N	Y	Y	Y
Family characteristics	N	N	N	N	N	Y	Y
Year dummies	N	N	N	N	N	N	Y
Province dummies	N	N	N	N	N	N	Y
Constant	3.759^***^ (0.219)	1.990^***^ (0.219)	2.004^***^ (0.205)	2.074^***^ (0.256)	2.057^***^ (0.211)	2.024^***^ (0.220)	1.690^***^ (0.237)
Observations	8964	8964	8934	8916	8460	8406	8406

*** indicate significance at the levels of 1, 5, and 10%, respectively.

### Robustness checks

We examine the robustness of physical activity's role in reducing the negative effects of depression among depressed individuals in the following five ways. First, we use the dummy variable representing “whether the life and work are often or frequently affected by depression” as the explained variable. This indicator is corresponding to *Problem*_*depression* from the same question in CGSS. Supplementary Table 5 shows that when this variable is used to characterize depression's adverse impacts, the estimated coefficients of physical exercise are also significantly negative at the 1% level, further proving the robustness of findings in the benchmark analysis. Second, we use a dummy variable “whether doing physical exercise per week” as the explanatory variable. Results of Supplementary Table 6 demonstrate that when using this indicator to reflect people's physical activity habits, it is also proved that exercise has a significant impact in reducing the adverse effects of depression. Third, we perform regressions using other statistical models, including the Ordinary Least Squares (OLS) and the Ordered Logit (Ologit) model. Supplementary Tables 7, 8 indicate that regardless of which model is applied and which variables are controlled in the regression, estimates of physical activity are all significantly negative at the 1% level.

Fourth, we further examine the explanatory power of physical activity on the explained variables using the machine learning method. Results in Supplementary Table 9 show that in all penalized machine learning models, physical activity consistently serves as the key indicator for predicting the explained variables. In Supplementary Figures 1, 2, the coefficients paths of independent variables illustrate that the explanatory power of physical activity is very robust. Fifth, we perform a placebo analysis of the results. Specifically, we randomly reallocate the *Physical*_*activity* variable in the sample and perform regressions using the generated new sample. Supplementary Figure 3 exhibits that estimates of physical activity in 1,000 such kind of new samples are all greater than that in the benchmark regression. Besides, their mean value is close to 0 and almost all the corresponding *P*-values are >0.1. This further proves that results in this paper are not affected by endogeneity caused by omitted random factors.

## Conclusion and discussion

This paper empirically investigates the role of physical activity in reducing the negative effects of depression among people with mental disorders. Empirical results show that physical exercise can help to mitigate depression's adverse consequences on work and life for depressed individuals. We find that the mechanism of this effect is that physical activity helps to reduce the severity of depression, enhance life satisfaction, improve mood, and make people have a better sense of purpose and meaning in life. Consequently, from the perspective of multidimensional subjective wellbeing, evaluative wellbeing, experienced wellbeing and eudaimonic wellbeing play mediating roles in the reduction of depression's adverse effects. Heterogeneity analysis shows that there are no significant gender differences in the health benefits of physical exercise, but the positive impact is more prominent for people with mental disorders who are younger and higher educated, with better health status, and live in urban areas. We also find that socioeconomic status plays an important moderating role. The health benefits of physical activity are greater for depressed people who have lower income, work in the secondary labor market, and have lower levels of social capital and assets. In addition, the instrumental variable approach is applied to identify the causal impact of physical activity, which further proves a significant effect of it based on tackling the endogeneity problem. Meanwhile, this paper uses different explanatory and explained variables, different regression models, as well as machine learning and placebo techniques to conduct robustness tests, all of which lend credence to above findings.

This study confirms the positive impact of physical activity on people with mental disorders. The existing research has generally discussed the relationship between physical exercise and mental health. For example, studies based on adolescents have found that physical exercise can alleviate depression caused by interpersonal problems (25), and higher levels of exercise have a positive effect on mental health (19, 26). At the same time, other studies have found that exercises help to reduce the frequency of smoking and develop a lifestyle conducive to both physical and mental health (15). When people are physically inactive, they are more likely to have physical diseases as well as psychological disorders (16, 17, 20). For example, because people worked at home for a long time during the COVID-19 epidemic, physical constraints of space reduce the exercise frequency and increase negative emotions as a result (40, 51). In addition, physical exercise can improve people's sense of happiness (28, 29). Research has also found that a healthy lifestyle brought by physical activities can help reduce the decline in abilities caused by aging (52). Regular exercise reduces the morbidity and improves the quality of life (53). However, it remains to be tested whether physical activity helps to improve the wellbeing of individuals already suffering from depression. Therefore, this paper extends the research on the relationship between exercise and mental health in the existing literature, demonstrating that it plays an important and positive role in alleviating depression's adverse effects on the work and life among depressed individuals. The more depressed individuals participate in physical activities, the less frequently they suffer from depression problems. This conclusion is robust to different explanatory and explained variables, various regression models, as well as machine learning and placebo tests.

In addition, as a typical manifestation of mental disorders, depression is strongly associated with lower living satisfaction, poorer overall health and even higher risk of death (2–4). Higher levels of recreational physical activity can help improve people's physical fitness and happiness in life (54). It is also found that lower levels of exercise frequency increase people's psychological distress (21, 22). In this paper, we support and expand the conclusion that exercises can reduce the mental burdens by reducing the severity of depression among those suffering from mental disorders. At the same time, based on the classification of subjective wellbeing in existing literature (46–48), this paper deepens the research on this issue. We further discover the mechanism of multidimensional subjective wellbeing in physical activity's impact on mental disorders. Specifically, this study reveals that exercises can reduce the negative effects of depression on people's work and life by enhancing life satisfaction, improving mood, and making them have a better sense of purpose and meaning in life. Depression and happiness are the two most important opposite emotions (55), and this paper demonstrates that exercises can reduce the negative effects of mental disorders by improving people's happiness.

At the same time, compared with previous studies that support the positive effects of exercises (19, 25, 26, 28, 29), we further systematically examine the heterogeneities in physical activity's benefits among different subgroups. Heterogeneity analysis shows that there is no significant gender difference in the health benefits of physical exercise, but its impact is more prominent for people with mental disorders who are younger and higher educated, with better health status, and live in urban areas. We also find that socioeconomic status plays an important moderating role. The health benefits of physical activity are greater for depressed people who have lower income, work in the secondary labor market, and have lower levels of social capital and assets. These findings not only enrich our understanding of the positive effects of physical activity, but also help us to better target our recommendations to specific subgroups to reduce depression's consequences on their work and life.

This research has important clinical implications for applying physical exercise to alleviate the adverse effects of depression on people's life and work. First, this paper proves the important role of physical activity in reducing depression levels and increasing the sense of wellbeing among people with mental disorders. This implies that the health benefits of exercise should be emphasized in clinical practice in addition to conventional treatment measures, such as antidepressants and psychotherapy. Second, the heterogeneity analysis results have important implications. For example, depressed patients who are younger, higher educated, in better physical condition, and with lower socioeconomic status should be advised more to gain greater health benefits from physical activity. The shortcomings of this paper mainly include two aspects. First, this paper uses the instrumental variable method to solve the endogeneity problem. Because this instrumental variable is only available for individuals with jobs, this approach results in sample loss. Therefore, randomized controlled experiments would be a better way to deal with endogeneity. Second, this paper uses a cross-sectional dataset, which is not as advantageous as longitudinal data in controlling individual fixed effects. Therefore, conducting randomized controlled experiments on physical activity among those with depression would be a very valuable research direction.

## Data availability statement

The data that support the findings of this study are available from Chinese General Social Survey (CGSS, http://cgss.ruc.edu.cn/English/Home.htm). Restrictions apply to the availability of these data, which were used under license for this study. Data are available from the authors with the permission of CGSS.

## Ethics statement

The studies involving human participants were reviewed and approved by the Institutional Review Board, Renmin University of China. The participants provided their written informed consent to participate in the survey.

## Author contributions

CL contributed to the conception and design of the study and performed the statistical analysis. YX generated the tables and figures, respectively, based on CL's analysis. CL and GN wrote the first draft of the manuscript. GN and QL worked on revisions of the manuscript. All authors provided critical feedback and approved the final submission.

## Funding

This work was supported by the Humanities and Social Science Research Project of the Ministry of Education of China [grant number 19YJC790055]; the Project of Natural Science Foundation of China [grant number 71973081]; the Project of Natural Science Foundation of Shandong Province, China [grant number ZR2020QG038]; the Project of Social Science Foundation of Shandong Province, China [grant number 19DJJJ08]; and the Project of Teaching Reform of Shandong University, Weihai [grant number Y2022007].

## Conflict of interest

The authors declare that the research was conducted in the absence of any commercial or financial relationships that could be construed as a potential conflict of interest.

## Publisher's note

All claims expressed in this article are solely those of the authors and do not necessarily represent those of their affiliated organizations, or those of the publisher, the editors and the reviewers. Any product that may be evaluated in this article, or claim that may be made by its manufacturer, is not guaranteed or endorsed by the publisher.

## Footnotes

1 Hukou is a system of household registration used in mainland China, mainly identifying a person as a rural or urban resident.

2 Working in-system in China refers to having jobs in Communist Party of China organizations, governments and state-owned corporations. Compared with out-of-system jobs, in-system jobs bring better social security and additional hidden benefits. Therefore, in-system is traditionally regarded as the primary labor market in China, while out-of-system means the secondary labor market.

3 The education level is classified from 1 to 13: 1-without any education, 2-kindergarten, 3-primary school, 4-junior high school, 5-vocational high school, 6-ordinary high school, 7-techinical secondary school, 8-technical high school, 9-junior college (adult education), 10-junior college (regular education), 11-undergraduate (adult education), 12-undergraduate (regular education), 13-postgraduate and above. Health status is based on the self-rated health levels from 1 to 5: 1-very unhealthy, 2-relatively unhealthy, 3-medium, 4-relatively healthy, 5-very healthy.

## References

[1] Rajabzadeh V Burn E Sajun SZ Suzuki M Bird VJ Priebe S. Understanding global mental health: a conceptual review. BMJ Glob Health. (2021) 6:e004631. 10.1136/bmjgh-2020-004631 33758013 PMC7993328

[2] Stein DJ Benjet C Gureje O Lund C Scott KM Poznyak V. Integrating mental health with other non-communicable diseases. BMJ. (2019) 364:l295. 10.1136/bmj.l295 30692081 PMC6348425

[3] Firth J Solmi M Wootton RE Vancampfort D Schuch FB Hoare E. A meta-review of “lifestyle psychiatry”: the role of exercise, smoking, diet and sleep in the prevention and treatment of mental disorders. World Psychiatry. (2020) 19:360–80. 10.1002/wps.20773 32931092 PMC7491615

[4] Xie Y Wu Z Sun L Zhou L Wang G Xiao L. The effects and mechanisms of exercise on the treatment of depression. Front Psychiatry. (2021) 12:705559. 10.3389/fpsyt.2021.705559 34803752 PMC8602192

[5] Figueroa CA Aguilera A. The need for a mental health technology revolution in the COVID-19 pandemic. Front Psychiatry. (2020) 11:523. 10.3389/fpsyt.2020.00523 32581891 PMC7283500

[6] He H Wu Q Hao Y Chen S Liu T Liao Y. Stigmatizing attitudes toward depression among male and female, medical and non-medical major college students. Front Psychol. (2021) 12:648059. 10.3389/fpsyg.2021.648059 34248746 PMC8267999

[7] United Nations. Transforming our world: the 2030 agenda for sustainable development (2015). https://sdgs.un.org/2030agenda

[8] Penninx BW Pine DS Holmes EA Reif A. Anxiety disorders. Lancet. (2021) 397:914–27. 10.1016/S0140-6736(21)00359-7 33581801 PMC9248771

[9] Stelmach R Kocher EL Kataria I Jackson-Morris AM Saxena S Nugent R. The global return on investment from preventing and treating adolescent mental disorders and suicide: a modelling study. BMJ Glob Health. (2022) 7:e007759. 10.1136/bmjgh-2021-007759 35705224 PMC9240828

[10] Lin Y Xie H Huang Z Zhang Q Wilson A Hou J. The mental health of transgender and gender non-conforming people in China: a systematic review. Lancet Public Health. (2021) 6:e954–69. 10.1016/S2468-2667(21)00236-X 34838199

[11] Tomlinson A Efthimiou O Boaden K New E Mather SE Salanti G. Side effect profile and comparative tolerability of 21 antidepressants in the acute treatment of major depression in adults: protocol for a network meta-analysis. Evid-Based Ment Health. (2019) 22:61–6. 10.1136/ebmental-2019-300087 30996028 PMC10270374

[12] Hieronymus F Lisinski A Eriksson E Østergaard SD. Do side effects of antidepressants impact efficacy estimates based on the Hamilton depression rating scale? A pooled patient-level analysis. Transl Psychiatry. (2021) 11:249. 10.1038/s41398-021-01364-0 33907188 PMC8079707

[13] Fibbins H Edwards L Morell R Lederman O Ward P Curtis J. Implementing an exercise physiology clinic for consumers within a community mental health service: a real-world evaluation. Front Psychiatry. (2021) 12:791125. 10.3389/fpsyt.2021.791125 34899443 PMC8651871

[14] Makhashvili N Javakhishvili JD Sturua L Pilauri K Fuhr DC Roberts B. The influence of concern about COVID-19 on mental health in the Republic of Georgia: a cross-sectional study. Global Health. (2020) 16:111. 10.1186/s12992-020-00641-9 33208153 PMC7672175

[15] Xia L Jiang F Rakofsky J Zhang Y Zhang K Liu T. Cigarette smoking, health-related behaviors, and burnout among mental health professionals in China: a nationwide survey. Front Psychiatry. (2020) 11:706. 10.3389/fpsyt.2020.00706 32765329 PMC7379885

[16] Cosco TD Wister A Riadi I Kervin L Best J Raina P. Reduced ability to engage in social and physical activity and mental health of older adults during the COVID-19 pandemic: longitudinal analysis from the Canadian longitudinal study on aging. Lancet. (2021) 398:S35. 10.1016/S0140-6736(21)02578-2 37771714 PMC10533203

[17] Hu S Tucker L Wu C Yang L. Beneficial effects of exercise on depression and anxiety during the Covid-19 pandemic: a narrative review. Front Psychiatry. (2020) 11:587557. 10.3389/fpsyt.2020.587557 33329133 PMC7671962

[18] Chen R Peng K Liu J Wilson A Wang Y Wilkinon MR. Interpersonal trauma and risk of depression among adolescents: the mediating and moderating effect of interpersonal relationship and physical exercise. Front Psychiatry. (2020) 11:194. 10.3389/fpsyt.2020.00194 32351408 PMC7174748

[19] Guddal MH Stensland SØ Småstuen MC Johnsen MB Zwart J-A Storheim K. Physical activity and sport participation among adolescents: associations with mental health in different age groups. Results from the Young-HUNT study: a cross-sectional survey. BMJ Open. (2019) 9:e028555. 10.1136/bmjopen-2018-028555 31488476 PMC6731817

[20] van Sluijs EMF Ekelund U Crochemore-Silva I Guthold R Ha A Lubans D. Physical activity behaviours in adolescence: current evidence and opportunities for intervention. Lancet. (2021) 398:429–42. 10.1016/S0140-6736(21)01259-9 34302767 PMC7612669

[21] Villani L Pastorino R Molinari E Anelli F Ricciardi W Graffigna G. Impact of the COVID-19 pandemic on psychological well-being of students in an Italian university: a web-based cross-sectional survey. Global Health. (2021) 17:39. 10.1186/s12992-021-00680-w 33823897 PMC8022300

[22] López-Bueno R Calatayud J Ezzatvar Y Casajús JA Smith L Andersen LL. Association between current physical activity and current perceived anxiety and mood in the initial phase of COVID-19 confinement. Front Psychiatry. (2020) 11:729. 10.3389/fpsyt.2020.00729 32793013 PMC7390883

[23] Nie Y Ma Y Wu Y Li J Liu T Zhang C. Association between physical exercise and mental health during the COVID-19 outbreak in China: a nationwide cross-sectional study. Front Psychiatry. (2021) 12:722448. 10.3389/fpsyt.2021.722448 34489763 PMC8418057

[24] Lokman JC Bockting CL. Pathways to depressive and anxiety disorders during and after the COVID-19 pandemic. Lancet Psychiatry. (2022) 9:531–3. 10.1016/S2215-0366(22)00152-3 35717953 PMC9212978

[25] Fancourt D Aughterson H Finn S Walker E Steptoe A. How leisure activities affect health: a narrative review and multi-level theoretical framework of mechanisms of action. Lancet Psychiatry. (2021) 8:329–39. 10.1016/S2215-0366(20)30384-9 33581775 PMC7613155

[26] Herbert C. Enhancing mental health, well-being and active lifestyles of university students by means of physical activity and exercise research programs. Front Public Health. (2022) 10:849093. 10.3389/fpubh.2022.849093 35548074 PMC9082407

[27] Oakman J Kinsman N Stuckey R Graham M Weale V. A rapid review of mental and physical health effects of working at home: how do we optimise health? BMC Public Health. (2020) 20:1825. 10.1186/s12889-020-09875-z 33256652 PMC7703513

[28] Lambert L Draper ZA Warren MA Joshanloo M Chiao EL Schwam A. Conceptions of happiness matter: relationships between fear and fragility of happiness and mental and physical wellbeing. J Happiness Stud. (2021) 23:535–60. 10.1007/s10902-021-00413-1

[29] King JE Jebeile H Garnett SP Baur LA Paxton SJ Gow ML. Physical activity based pediatric obesity treatment, depression, self-esteem and body image: a systematic review with meta-analysis. Ment Health Phys Act. (2020) 19:100342. 10.1016/j.mhpa.2020.100342 32020780

[30] Castellanos-García P Lera-López F Sánchez-Santos JM. Light, moderate and vigorous physical activities: new insights into a virtuous circle with happiness. Eur J Sport Sci. (2022). 10.1080/17461391.2022.2089053. [Epub ahead of print]. 35695097

[31] An HY Chen W Wang CW Yang HF Huang WT Fan SY. The relationships between physical activity and life satisfaction and happiness among young, middle-aged, and older adults. Int J Environ Res Public Health. (2020) 17:4817. 10.3390/ijerph17134817 32635457 PMC7369812

[32] Kuettel A Pedersen AK Larsen CH. To flourish or languish, that is the question: exploring the mental health profiles of Danish elite athletes. Psychol Sport Exerc. (2021) 52:101837. 10.1016/j.psychsport.2020.101837

[33] Carriedo A Cecchini JA Fernandez-Rio J Méndez-Giménez A. COVID-19, psychological well-being and physical activity levels in older adults during the nationwide lockdown in Spain. Am J Geriatr Psychiatry. (2020) 28:1146–55. 10.1016/j.jagp.2020.08.007 32919872 PMC7443087

[34] Lee J Kim J Chow A Piatt JA. Different levels of physical activity, physical health, happiness, and depression among older adults with diabetes. Gerontol Geriatr Med. (2021) 7:233372142199562. 10.1177/2333721421995623 33763506 PMC7944526

[35] Yu DJ Yu AP Leung CK Chin EC Fong DY Cheng CP. Comparison of moderate and vigorous walking exercise on reducing depression in middle-aged and older adults: a pilot randomized controlled trial. Eur J Sport Sci. (2022) 10.1080/17461391.2022.2079424. [Epub ahead of print]. 35579606

[36] Jiang W Luo J Guan H. Gender difference in the relationship of physical activity and subjective happiness among Chinese university students. Front Psychol. (2021) 12:800515. 10.3389/fpsyg.2021.800515 34950093 PMC8688753

[37] Chang J Chen Y Liu C Yong L Yang M Zhu W Wang J Yan J. Effect of square dance exercise on older women with mild mental disorders. Front Psychiatry. (2021) 12:699778. 10.3389/fpsyt.2021.699778 34393860 PMC8357995

[38] Yu G Song Y. What affects sports participation and life satisfaction among urban residents? The role of self-efficacy and motivation. Front Psychol. (2022) 13:884953. 10.3389/fpsyg.2022.884953 35572238 PMC9096906

[39] Matei R Ginsborg J. Physical activity, sedentary behavior, anxiety, and pain among musicians in the United Kingdom. Front Psychol. (2020) 11:560026. 10.3389/fpsyg.2020.560026 33424675 PMC7793824

[40] Lyttelton T Zang E Musick K. Gender differences in telecommuting and implications for inequality at home and work. SSRN Electr J. (2020) 1–51. 10.2139/ssrn.3645561

[41] Yarar EZ Roestorf A Spain D Howlin P Bowler D Charlton R. Aging and autism: Do measures of autism symptoms, co-occurring mental health conditions, or quality of life differ between younger and older autistic adults? Autism Res. (2022) 15:1482–94. 10.1002/aur.2780 35790084

[42] Uljarević M Hedley D Rose-Foley K Magiati I Cai RY Dissanayake C. Anxiety and depression from adolescence to old age in autism spectrum disorder. J Autism Dev Disord. (2019) 50:3155–65. 10.1007/s10803-019-04084-z 31190198

[43] Wang W Wang M Hu Q Wang P Lei L Jiang S. Upward social comparison on mobile social media and depression: the mediating role of envy and the moderating role of marital quality. J Affect Disorders. (2020) 270:143–9. 10.1016/j.jad.2020.03.173 32339106

[44] Gray TD Hawrilenko M Cordova JV. Randomized controlled trial of the marriage checkup: depression outcomes. J Marital Fam Ther. (2019) 46:507–22. 10.1111/jmft.12411 31584721

[45] Hentges RF Graham SA Fearon P Tough S Madigan S. The chronicity and timing of prenatal and antenatal maternal depression and anxiety on child outcomes at age 5. Depress Anxiety. (2020) 37:576–86. 10.1002/da.23039 32419282

[46] Martín-María N Lara E Cabello M Olaya B Haro JM Miret M. To be happy and behave in a healthier way. A longitudinal study about gender differences in the older population. Psychol Health. (2021). 10.1080/08870446.2021.1960988. [Epub ahead of print]. 34353185

[47] Deci EL Ryan RM. Hedonia, eudaimonia, and well-being: an introduction. J Happiness Stud. (2006) 9:1–11. 10.1007/s10902-006-9018-1

[48] Ryan RM Deci EL. On happiness and human potentials: a review of research on hedonic and eudaimonic well-being. Annu Rev Psychol. (2001) 52:141–66. 10.1146/annurev.psych.52.1.141 11148302

[49] Mihaylov E Tijdens KG. Measuring the routine and non-routine task content of 427 four-digit ISCO-08 occupations. In: Tinbergen Institute Discussion Paper. Amsterdam: Tinbergen Institute (2019) TI 2019-035/V. 10.2139/ssrn.3389681

[50] Acemoglu D Restrepo P. Robots and jobs: evidence from US labor markets. J Polit Econ. (2020) 128:2188–244. 10.1086/705716

[51] Schifano S Clark AE Greiff S Vögele C D'Ambrosio C. Well-being and working from home during COVID-19. Inform Technol Peopl. (2021) 1–19. 10.1108/ITP-01-2021-0033

[52] Wagner M Grodstein F Proust-Lima C Samieri C. Long-term trajectories of body weight, diet, and physical activity from midlife through late life and subsequent cognitive decline in women. Am J Epidemiol. (2020) 189:305–13. 10.1093/aje/kwz262 31781745 PMC7443200

[53] Semaan S Dewland TA Tison GH Nah G Vittinghoff E Pletcher MJ. Physical activity and atrial fibrillation: data from wearable fitness trackers. Heart Rhythm. (2020) 17:842–6. 10.1016/j.hrthm.2020.02.013 32354448 PMC8580729

[54] Lin YT Chen M Ho CC Lee TS. Relationships among leisure physical activity, sedentary lifestyle, physical fitness, and happiness in adults 65 years or older in Taiwan. Int J Env Res Public Health. (2020) 17:5235. 10.3390/ijerph17145235 32698473 PMC7400288

[55] Yildirim M Balahmar NB. Adaptation and validation of the Arabic version of the short depression-happiness scale. Curr Psychol. (2020) 41:7024–31. 10.1007/s12144-020-01214-0

