# Supplementary material for: Health benefits of physical activity for people with mental disorders: From the perspective of multidimensional subjective wellbeing
Source: Front Psychiatry. 2022 Nov 17;13:1050208. doi: 10.3389/fpsyt.2022.1050208 (PMC9712743; doi:10.3389/fpsyt.2022.1050208)
Supplement: Supplementary file 1 [file Data_Sheet_1.pdf]

Supplementary Material

Supplementary Table 1. Summary Statistics

Variable	Description	Obs.	Mean	Std. Dev.	Min.	Max.
Dependent Variables						
Problem_depression	1-5 levels	25325	2.077	1.130	1	5
Whether having depression problems	Yes=1, No=0	25325	0.298	0.458	0	1
Explanatory Variable						
Physical_activity	Frequency of physical activities per week	25216	2.267	3.233	0	21
Whether doing physical activities	Yes=1, No=0	25216	0.466	0.499	0	1
Control Variables						
Demographic Characteristics						
Age	Age	25369	51.423	16.911	18	118
Age_squared	Squared term of age	25369	2930.297	1760.003	324	13924
Whether female	Yes=1, No=0	25369	0.531	0.499	0	1
Human Capital Characteristics						
Education level	1-13 levels	25355	0.111	3.314	0	1
Whether migrants	Yes=1, No=0	25298	0.139	0.346	0	1
Social Characteristics						
Whether Hukou in urban	Yes=1, No=0	25295	0.373	0.484	0	1
Whether ethnic minorities	Yes=1, No=0	25369	0.074	0.262	0	1
Whether religious believer	Yes=1, No=0	25369	0.105	0.307	0	1
Whether CPC member	Yes=1, No=0	25336	0.111	0.315	0	1
Working Characteristics						
ln_Income	Logarithm of personal total income (RMB)	24008	8.363	3.862	0	16.117
Whether working in the system	Yes=1, No=0	25236	0.062	0.242	0	1
Whether having pension	Yes=1, No=0	25308	0.740	0.439	0	1
Whether having medical insurance	Yes=1, No=0	25333	0.928	0.258	0	1
Family Characteristics						
Whether married	Yes=1, No=0	25369	0.752	0.432	0	1
Family size	Number of members in the family	25336	2.812	1.492	1	44
Number of children	Number of children in the family	25320	1.698	1.312	0	32
Number of houses	Number of houses in the family	25086	1.099	0.653	0	15
Year dummies						
Province dummies						

Note: The education level is classified from 1 to 13: 1-without any education, 2-kindergarten, 3-primary school, 4-junior high school, 5-vocational high school, 6-ordinary high school, 7-techinical secondary school, 8-technical high school, 9-junior college (adult education), 10-junior college (regular education), 11-undergraduate (adult education), 12-undergraduate (regular education), 13-postgraduate and above.

Supplementary Table 2. The First Stage Regression of Instrumental Variable Methods

**Model**	**First Stage**
**Model**	**Physical_activity**
Physical_activity	
Automation	0.463*** (0.138)
Constant	0.813* (0.420)
Controls	Yes
Year dummies	Yes
Province dummies	Yes
Observations	8406

Supplementary Table 3. Instrumental Variable Estimations Using 2SLS

Model<br>Variable	(1) 2SLS Problem_ depression	(2) 2SLS Problem_ depression	(3) 2SLS Problem_ depression	(4) 2SLS Problem_ depression	(5) 2SLS Problem_ depression	(6) 2SLS Problem_ depression	(7) 2SLS Problem_ depression
Physical_ activity	-0.846*** (0.115)	-0.450*** (0.077)	-0.437*** (0.082)	-0.473*** (0.128)	-0.384*** (0.110)	-0.402*** (0.122)	-0.378*** (0.138)
Age		0.020*** (0.007)	0.019*** (0.007)	0.019** (0.008)	0.018** (0.007)	0.025*** (0.008)	0.020*** (0.008)
Age_squared		0.000 (0.000)	0.000 (0.000)	0.000 (0.000)	0.000 (0.000)	-0.000 (0.000)	0.000 (0.000)
Whether female		0.043 (0.038)	0.045 (0.038)	0.042 (0.044)	0.045 (0.036)	0.049 (0.036)	0.062* (0.036)
Education level			0.108 (0.071)	0.066 (0.065)	0.033 (0.055)	0.032 (0.058)	0.058 (0.056)
Whether migrants			-0.165*** (0.049)	-0.160*** (0.053)	-0.128*** (0.048)	-0.143*** (0.049)	-0.082 (0.050)
Whether Hukou in urban				0.053 (0.086)	0.002 (0.063)	0.002 (0.066)	0.030 (0.064)
Whether ethnic minorities				-0.066 (0.092)	-0.019 (0.079)	-0.026 (0.083)	-0.098 (0.087)
Whether religious believer				-0.018 (0.062)	-0.002 (0.057)	-0.005 (0.059)	0.032 (0.058)
Whether CPC member				0.104 (0.103)	0.058 (0.085)	0.080 (0.091)	0.072 (0.094)
ln_Income					-0.020** (0.008)	-0.018** (0.009)	-0.013 (0.009)
Whether working in the system					0.149* (0.076)	0.157* (0.082)	0.139 (0.085)
Whether having pension					-0.037 (0.040)	-0.040 (0.042)	0.001 (0.040)
Whether having medical insurance					0.081 (0.069)	0.105 (0.072)	0.090 (0.068)
Whether married						-0.095** (0.047)	-0.078* (0.045)
Family size						-0.013 (0.011)	-0.023** (0.011)
Number of children						0.001 (0.020)	-0.003 (0.019)
Number of houses						-0.017 (0.029)	-0.005 (0.028)
Year dummies	No	No	No	No	No	No	Yes
Province dummies	No	No	No	No	No	No	Yes
Constant	3.759*** (0.219)	1.990*** (0.219)	2.004*** (0.205)	2.074*** (0.256)	2.057*** (0.211)	2.024*** (0.220)	1.690*** (0.237)
Observations	8964	8964	8934	8916	8460	8406	8406

Supplementary Table 4. Using Other Instrumental Variable Methods

**Model**	**(1)<br>2SLS<br>Second Stage**	**(2)<br>LIML<br>Second Stage**	**(3)<br>GMM<br>Second Stage**	**(4)<br>IGMM<br>Second Stage**
**Model**	**Problem_<br>depression**	**Problem_<br>depression**	**Problem_<br>depression**	**Problem_<br>depression**
Physical_activity	-0.378***	-0.378***	-0.378***	-0.378***
	(0.138)	(0.138)	(0.138)	(0.138)
Automation				
Constant	1.690***	1.690***	1.690***	1.690***
	(0.237)	(0.237)	(0.237)	(0.237)
Controls	Yes	Yes	Yes	Yes
Year dummies	Yes	Yes	Yes	Yes
Province dummies	Yes	Yes	Yes	Yes
Observations	8406	8406	8406	8406

Supplementary Table 5. Robustness Tests: Using Another Dependent Indicator

Model<br>Variable	(1) Probit Whe_depression_problem	(2) Probit Whe_depression_problem	(3) Probit Whe_depression_problem	(4) Probit Whe_depression_problem	(5) Probit Whe_depression_problem	(6) Probit Whe_depression_problem	(7) Probit Whe_depression_problem
Physical_activity	-0.047*** (0.004)	-0.047*** (0.004)	-0.045*** (0.004)	-0.037*** (0.004)	-0.036*** (0.004)	-0.034*** (0.004)	-0.032*** (0.004)
Age		0.051*** (0.004)	0.043*** (0.004)	0.043*** (0.004)	0.047*** (0.004)	0.053*** (0.004)	0.051*** (0.005)
Age_squared		-0.000*** (0.000)	-0.000*** (0.000)	-0.000*** (0.000)	-0.000*** (0.000)	-0.000*** (0.000)	-0.000*** (0.000)
Whether female		0.165*** (0.020)	0.162*** (0.020)	0.147*** (0.021)	0.105*** (0.022)	0.099*** (0.022)	0.115*** (0.022)
Education level			-0.219*** (0.043)	-0.032 (0.045)	0.014 (0.048)	0.036 (0.049)	0.071 (0.049)
Whether migrants			-0.204*** (0.034)	-0.185*** (0.035)	-0.179*** (0.036)	-0.185*** (0.037)	-0.107*** (0.039)
Whether Hukou in urban				-0.288*** (0.023)	-0.229*** (0.025)	-0.223*** (0.026)	-0.160*** (0.028)
Whether ethnic minorities				0.220*** (0.039)	0.223*** (0.040)	0.220*** (0.040)	0.093* (0.048)
Whether religious believer				0.010 (0.033)	0.010 (0.035)	0.007 (0.035)	0.057 (0.037)
Whether CPC member				-0.189*** (0.038)	-0.155*** (0.039)	-0.141*** (0.040)	-0.149*** (0.040)
ln_Income					-0.030*** (0.003)	-0.030*** (0.003)	-0.027*** (0.003)
Whether working in the system					-0.033 (0.054)	-0.033 (0.055)	-0.050 (0.055)
Whether having pension					-0.016 (0.026)	-0.008 (0.027)	0.011 (0.027)
Whether having medical insurance					0.064 (0.044)	0.077* (0.045)	0.056 (0.045)
Whether married						-0.105*** (0.028)	-0.098*** (0.029)
Family size						-0.020** (0.008)	-0.020** (0.008)
Number of children						0.033*** (0.010)	0.019* (0.010)
Number of houses						-0.091*** (0.020)	-0.076*** (0.020)
Constant	-0.226*** (0.012)	-2.414*** (0.102)	-2.131*** (0.106)	-2.117*** (0.108)	-2.030*** (0.117)	-1.951*** (0.122)	-2.248*** (0.136)
Year dummies	No	No	No	No	No	No	Yes
Province	No	No	No	No	No	No	Yes

dummies							
Observations	17840	17840	17786	17740	16721	16559	16559
Pseudo R^2	0.009	0.116	0.119	0.129	0.132	0.133	0.149

Supplementary Table 6. Robustness Tests: Using Another Physical Activity Indicator

Model<br>Variable	(1) Oprobit Problem_ depression	(2) Oprobit Problem_ depression	(3) Oprobit Problem_ depression	(4) Oprobit Problem_ depression	(5) Oprobit Problem_ depression	(6) Oprobit Problem_ depression	(7) Oprobit Problem_ depression
Whether participating in physical activity	-0.409*** (0.016)	-0.300*** (0.016)	-0.284*** (0.016)	-0.231*** (0.017)	-0.216*** (0.017)	-0.214*** (0.018)	-0.196*** (0.018)
Age		0.036*** (0.003)	0.030*** (0.003)	0.030*** (0.003)	0.036*** (0.003)	0.043*** (0.003)	0.036*** (0.003)
Age_squared		-0.000*** (0.000)	-0.000*** (0.000)	-0.000*** (0.000)	-0.000*** (0.000)	-0.000*** (0.000)	-0.000*** (0.000)
Whether female		0.113*** (0.016)	0.111*** (0.016)	0.101*** (0.016)	0.063*** (0.017)	0.058*** (0.017)	0.076*** (0.018)
Education level			-0.137*** (0.029)	-0.003 (0.031)	0.016 (0.033)	0.023 (0.034)	0.063* (0.034)
Whether migrants			-0.237*** (0.026)	-0.233*** (0.026)	-0.214*** (0.028)	-0.225*** (0.028)	-0.123*** (0.030)
Whether Hukou in urban				-0.222*** (0.018)	-0.173*** (0.019)	-0.173*** (0.020)	-0.105*** (0.021)
Whether ethnic minorities				0.136*** (0.032)	0.134*** (0.033)	0.134*** (0.033)	0.057 (0.038)
Whether religious believer				-0.055** (0.027)	-0.051* (0.029)	-0.048* (0.029)	0.011 (0.030)
Whether CPC member				-0.187*** (0.030)	-0.153*** (0.031)	-0.134*** (0.031)	-0.129*** (0.031)
ln_Income					-0.027*** (0.002)	-0.027*** (0.002)	-0.024*** (0.002)
Whether working in the system					0.014 (0.038)	0.015 (0.038)	0.002 (0.039)
Whether having pension					-0.044** (0.021)	-0.036* (0.021)	-0.006 (0.021)
Whether having medical insurance					0.064* (0.035)	0.089** (0.036)	0.073** (0.036)
Whether married						-0.115*** (0.022)	-0.111*** (0.023)
Family size						-0.028*** (0.006)	-0.028*** (0.006)
Number of children						0.026*** (0.008)	0.016** (0.008)
Number of houses						-0.089*** (0.015)	-0.075*** (0.015)
Year dummies	No	No	No	No	No	No	Yes
Province dummies	No	No	No	No	No	No	Yes

Observations	17840	17840	17786	17740	16721	16559	16559
Pseudo R^2	0.012	0.060	0.062	0.066	0.068	0.070	0.079

Supplementary Table 7. Robustness Tests: Using Ordinary Least Squares Model

Model<br>Variable	(1) OLS Problem_ depression	(2) OLS Problem_ depression	(3) OLS Problem_ depression	(4) OLS Problem_ depression	(5) OLS Problem_ depression	(6) OLS Problem_ depression	(7) OLS Problem_ depression
Physical_ activity	-0.046*** (0.003)	-0.042*** (0.003)	-0.041*** (0.003)	-0.034*** (0.003)	-0.033*** (0.003)	-0.032*** (0.003)	-0.030*** (0.003)
Age		0.031*** (0.002)	0.025*** (0.003)	0.025*** (0.003)	0.032*** (0.003)	0.039*** (0.003)	0.035*** (0.003)
Age_squared		-0.000** (0.000)	-0.000 (0.000)	-0.000 (0.000)	-0.000** (0.000)	-0.000*** (0.000)	-0.000*** (0.000)
Whether female		0.110*** (0.016)	0.108*** (0.016)	0.095*** (0.016)	0.054*** (0.017)	0.048*** (0.017)	0.063*** (0.017)
Education level			-0.157*** (0.023)	-0.006 (0.025)	0.022 (0.026)	0.026 (0.027)	0.061** (0.027)
Whether migrants			-0.210*** (0.022)	-0.205*** (0.022)	-0.182*** (0.023)	-0.193*** (0.024)	-0.102*** (0.025)
Whether Hukou in urban				-0.242*** (0.017)	-0.187*** (0.018)	-0.183*** (0.018)	-0.122*** (0.019)
Whether ethnic minorities				0.155*** (0.032)	0.151*** (0.033)	0.149*** (0.032)	0.070* (0.037)
Whether religious believer				-0.040 (0.027)	-0.035 (0.028)	-0.034 (0.028)	0.017 (0.029)
Whether CPC member				-0.186*** (0.027)	-0.148*** (0.028)	-0.128*** (0.029)	-0.126*** (0.028)
ln_Income					-0.029*** (0.002)	-0.029*** (0.002)	-0.026*** (0.002)
Whether working in the system					-0.008 (0.032)	-0.006 (0.032)	-0.020 (0.033)
Whether having pension					-0.039* (0.020)	-0.031 (0.020)	-0.004 (0.020)
Whether having medical insurance					0.050 (0.034)	0.073** (0.034)	0.058* (0.034)
Whether married						-0.124*** (0.022)	-0.120*** (0.022)
Family size						-0.027*** (0.006)	-0.026*** (0.006)
Number of children						0.032*** (0.008)	0.022*** (0.008)
Number of houses						-0.075*** (0.013)	-0.061*** (0.012)
Year dummies	No	No	No	No	No	No	Yes
Province dummies	No	No	No	No	No	No	Yes
Constant	2.423*** (0.011)	0.919*** (0.057)	1.161*** (0.061)	1.193*** (0.062)	1.244*** (0.071)	1.295*** (0.077)	1.030*** (0.086)
Observations	17840	17840	17786	17740	16721	16559	16559
Adjusted R^2	0.016	0.156	0.161	0.175	0.182	0.186	0.206

Supplementary Table 8. Robustness Tests: Using Ordered-Logit Model

Model<br>Variable	(1) Ologit Problem_ depression	(2) Ologit Problem_ depression	(3) Ologit Problem_ depression	(4) Ologit Problem_ depression	(5) Ologit Problem_ depression	(6) Ologit Problem_ depression	(7) Ologit Problem_ depression
Physical_ activity	-0.071***	-0.072***	-0.070***	-0.058***	-0.057***	-0.056***	-0.053***
	(0.005)	(0.005)	(0.005)	(0.005)	(0.005)	(0.005)	(0.005)
Age		0.058***	0.047***	0.047***	0.059***	0.071***	0.066***
		(0.005)	(0.005)	(0.005)	(0.005)	(0.006)	(0.006)
Age_squared		-0.000***	-0.000	-0.000	-0.000***	-0.000***	-0.000***
		(0.000)	(0.000)	(0.000)	(0.000)	(0.000)	(0.000)
Whether female		0.211***	0.208***	0.191***	0.126***	0.114***	0.146***
		(0.028)	(0.028)	(0.028)	(0.030)	(0.030)	(0.030)
Education level			-0.279***	-0.032	0.006	0.019	0.091
			(0.048)	(0.051)	(0.055)	(0.057)	(0.057)
Whether migrants			-0.415***	-0.412***	-0.380***	-0.403***	-0.218***
			(0.044)	(0.045)	(0.048)	(0.048)	(0.051)
Whether Hukou in urban				-0.396***	-0.309***	-0.303***	-0.180***
				(0.031)	(0.033)	(0.034)	(0.036)
Whether ethnic minorities				0.251***	0.249***	0.249***	0.098
				(0.056)	(0.058)	(0.058)	(0.067)
Whether religious believer				-0.084*	-0.078	-0.073	0.029
				(0.048)	(0.050)	(0.051)	(0.053)
Whether CPC member				-0.318***	-0.254***	-0.222***	-0.211***
				(0.051)	(0.053)	(0.054)	(0.054)
ln_Income					-0.046***	-0.046***	-0.040***
					(0.004)	(0.004)	(0.004)
Whether working in the system					0.002	0.004	-0.022
					(0.063)	(0.063)	(0.065)
Whether having pension					-0.085**	-0.071**	-0.018
					(0.036)	(0.036)	(0.037)
Whether having medical insurance					0.114*	0.160**	0.129**
					(0.061)	(0.062)	(0.063)
Whether married						-0.190***	-0.180***
						(0.038)	(0.039)
Family size						-0.046***	-0.046***
						(0.011)	(0.011)
Number of children						0.052***	0.034**
						(0.013)	(0.013)
Number of houses						-0.162***	-0.134***
						(0.027)	(0.026)
Year dummies	No	No	No	No	No	No	Yes
Province dummies	No	No	No	No	No	No	Yes
Observations	17840	17840	17786	17740	16721	16559	16559
Pseudo R^2	0.005	0.058	0.061	0.066	0.068	0.070	0.080

Supplementary Table 9. Robustness Tests: Using Panelized Machine Learning Methods

Model<br><br>Variable	(1)<br>Lasso<br>(10-fold CV)<br>Problem_<br>depression	(2)<br>Lasso<br>(20-fold CV)<br>Problem_<br>depression	(3)<br>Ridge<br>(10-fold CV)<br>Problem_<br>depression	(4)<br>Ridge<br>(20-fold CV)<br>Problem_<br>depression	(5)<br>Elastic Net<br>(10-fold CV)<br>Problem_<br>depression	(6)<br>Elastic Net<br>(20-fold CV)<br>Problem_<br>depression
Physical_activity	-0.0302	-0.0302	-0.0290	-0.0290	-0.0302	-0.0302
No. of nonzero coefficients	46	46	48	48	46	46
λ	0.0003	0.0002	0.0420	0.0420	0.0003	0.0002
Out-of-sample R^2	0.2029	0.2036	0.2013	0.2019	0.2029	0.2036
CV mean prediction error	1.0322	1.0313	1.0343	1.0335	1.0322	1.0313
α					1	1
Observations	16559	16559	16559	16559	16559	16559

Explanation on the Panelized Machine Learning Methods: Since the influence of physical activity on mental disorders has not been paid attention to in the existing literature, we further test whether exercises have a strong explanatory power for mental disorders compared with other factors that have been discussed in the literature. First, we perform the analysis using Lasso Linear regression, applying 10-fold and 20-fold cross-validation to obtain the optimal penalty λ. When the penalty is 0, it would be the OLS estimation in the benchmark regression. As shown in Columns (1) - (2) of Supplementary Table 9, based on the 10-fold and 20-fold cross-validation methods, we obtain the optimal penalties of 0.0003 and 0.0002, respectively. Under the optimal λ, the two models both have 46 non-zero independent variables including physical exercise, and the estimated values of physical activities are negative, proving that physical activity is a necessary indicator for predicting the explained variable.

The estimation results obtained by the Ridge and Elastic Net models are consistent with that using Lasso. Here in this research, the Elastic Net model under the optimal α is equivalent to Lasso. The results in Supplementary Table 9 show that in all penalized machine learning models, physical activity consistently serves as the key indicator for predicting the explained variables. In Supplementary Figures 1 and 2, the coefficients paths of independent variables illustrate that the explanatory power of physical activity is very robust. These results prove that physical exercise is a very important and robust factor to predict mental disorders' effects compared to other variables.

Supplementary Figure 1. Coefficient Paths in the Lasso/Elastic Net Model

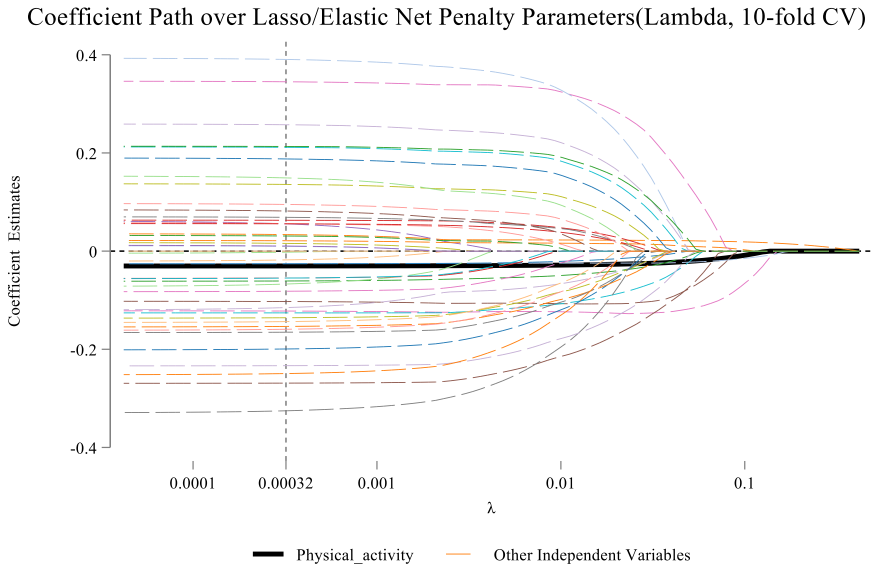

Supplementary Figure 2. Coefficient Paths in the Ridge Model

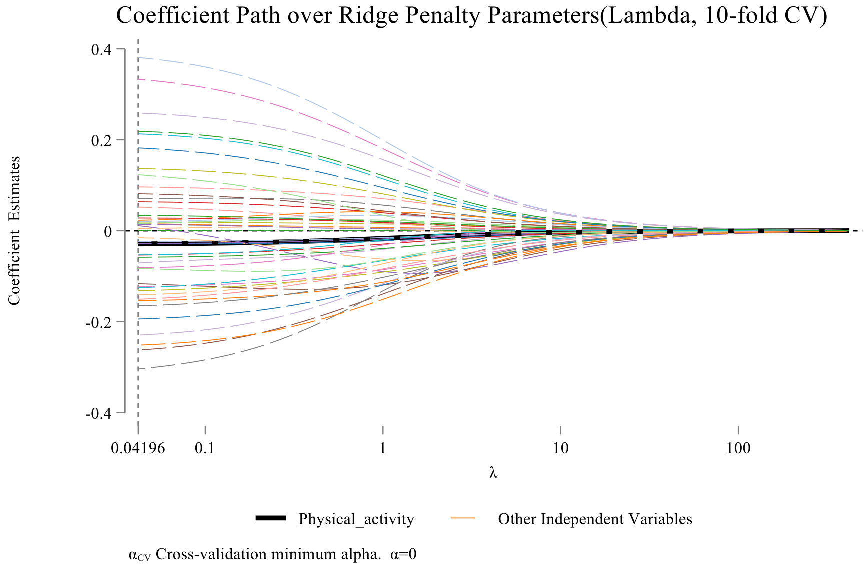

Supplementary Figure 3. Placebo Test

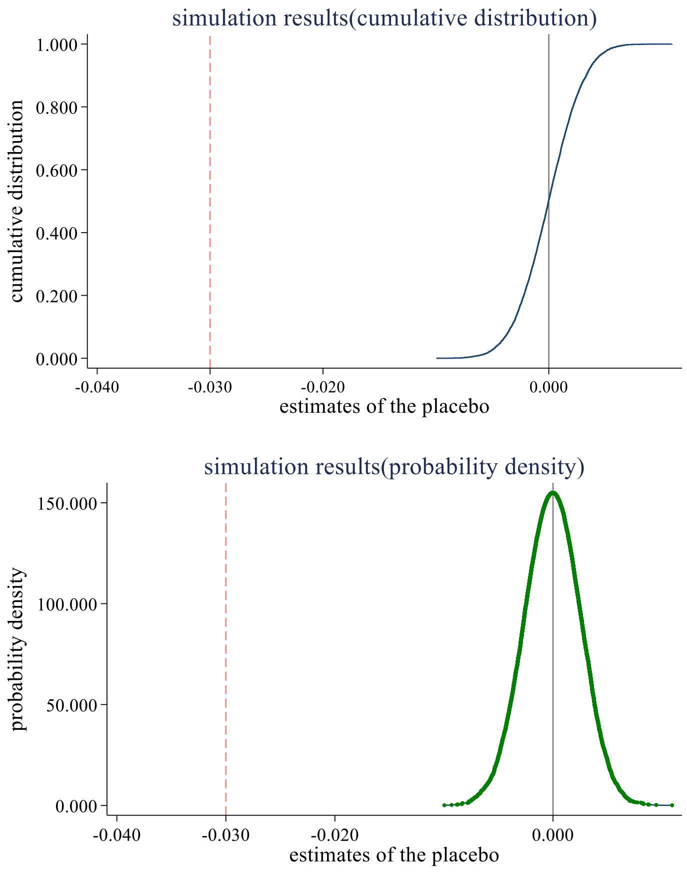

Supplementary Introduction of the Data

This paper uses a nationally representative observational dataset, which is the Chinese General Social Survey (CGSS). CGSS is in the world General Social Survey family, jointly carried out by Renmin University of China and Hong Kong University of Science and Technology. The sampling of CGSS is based on the multi-stage stratified design. The sampling stages are as follows: (1) PSUs are county-level units and there are 2762 PSUs in the sampling frame; (2) SSUs are community-level units (villages [cun] and neighborhood committees [ju wei hui]); (3) in selected SSU, 25 households (TSUs) are sampled with PPS method; (4) one eligible person aged 18-above is selected from each sampled household to serve as the representative. There are 43 Municipalities directly under the Chinese central government, provincial capital cities, and vice provincial cities in China. Comprehensive ranking by GDP, FDI and Education Level to these cities, the top 5 is Beijing, Shanghai, Tianjin, Guangzhou, and Shenzhen. CGSS treats these 5 cities as self-representative stratum. This stratum consists of 67 PSUs. The rest 2695 PSUs are comprehensively ranked with GDP per capital, urbanization rate, and population density and then are equally classified into 50 strata. Within each stratum, 2 PSUs will be selected with PPS method. In each selected PSU, 4 communities are sampled with PPS method. There are 80 communities in self-representative stratum and 400 communities in the rest 50 strata.

The national Survey Research Center at Renmin University of China (NSRC) organized Chinese Social Survey Network (CSSN), including 49 universities and provincial social science academies. Members of CSSN undertake the survey of the CGSS in their own provinces. The CGSS uses Computer Aided Personal Interviewing, and the average interview time is about one and a half hours. The CGSS has a set of strict quality control procedures, which cover pre-fieldwork, in-fieldwork, and after-fieldwork states. In pre-fieldwork stage, all supervisors must receive 40 hours training and finish 4 experimental interviews; all interviewers must receive 25 hours training and finish 3 experimental interviews. In in-fieldwork stage, all interviewers will be accompanied to interview by supervisor at least once. And the finished questionnaires must be 100% on site check and supervisors must do 40% the second day back interviewing. And in post-fieldwork stage, all interviews must 100% mail back interview and 40% telephone interview. In data input and coding stage, the data must be double input and double coding and there are several supervisors to check the double input and coding validation process.

The CGSS aims to collect quantitative data about (1) measures of social structure, its stability and change, (2) measures of quality of life, objective and subjective, and (3) measures of underlying mechanisms linking social structure and quality of life. The questionnaire of CGSS composes three kinds of modules: core module, topic module (rotation module), and additional module. The core module is annually repeated, which includes 152 variables. There are two kinds of the CGSS core module variables. One is the standard background variables, which include 71 variables. Another is the variables to trace social change trends. They are the rest 81 variables. The core module of the CGSS has 11 dimensions: Social demographic, health, lifestyle, migration, social attitude, class identity, political attitude and behavior, cognitive ability, labor market participation, social welfare, and family. The topic modules will be rotated every five years. There are one or two topic modules in the annual questionnaire. The topic modules aim to address important social issues. The additional modules include EASS module, ISSP module, and other ad hoc one-time modules. Some proposed topic modules also might be used as one-time additional modules. Questions in core module and topic modules will be asked to all respondents. Questions in additional modules only have one-third or a half chance to be asked.

Above information is taken from http://cgss.ruc.edu.cn/English/Home.htm.
